# TREND: a platform for exploring protein function in prokaryotes based on phylogenetic, domain architecture and gene neighborhood analyses

**DOI:** 10.1093/nar/gkaa243

**Published:** 2020-04-13

**Authors:** Vadim M Gumerov, Igor B Zhulin

**Affiliations:** Department of Microbiology and Translational Data Analytics Institute, The Ohio State University, Columbus, OH, USA; Department of Microbiology and Translational Data Analytics Institute, The Ohio State University, Columbus, OH, USA

## Abstract

Key steps in a computational study of protein function involve analysis of (i) relationships between homologous proteins, (ii) protein domain architecture and (iii) gene neighborhoods the corresponding proteins are encoded in. Each of these steps requires a separate computational task and sets of tools. Currently in order to relate protein features and gene neighborhoods information to phylogeny, researchers need to prepare all the necessary data and combine them by hand, which is time-consuming and error-prone. Here, we present a new platform, TREND (**tr**ee-based **e**xploration of **n**eighborhoods and **d**omains), which can perform all the necessary steps in automated fashion and put the derived information into phylogenomic context, thus making evolutionary based protein function analysis more efficient. A rich set of adjustable components allows a user to run the computational steps specific to his task. TREND is freely available at http://trend.zhulinlab.org.

## INTRODUCTION

In prokaryotes, genes that encode functionally linked (e.g. involved in the same biological pathway) proteins are often organized in gene clusters (1). Efficient regulation of transcription employs the same principle organizing genes in operons. Gene order in clusters/operons is often conserved especially in more closely related species (1). Operons that encode subunits of highly conserved multi-protein complexes, such as ribosomes, are conserved even in distantly related bacterial genomes and even between archaea and bacteria (2). Gene order conservation can be efficiently utilized in functional prediction of uncharacterized and poorly characterized proteins (3,4). Arrangement of constituent domains (domain architecture) to a large extent determines the function of a protein (5). Globular protein domains are structurally compact, independently folding units (6). Individual domains can expand their functional repertoire in a number of ways, including residue mutations, changes in the core fold of a domain that change ligand binding properties, altered interaction partners and loop extensions (7). Altered combination of domains is a source of functional changes in proteins and identification of complete domain architecture is an efficient instrument for their functional characterization (8). A protein sequence-based phylogenetic tree groups the proteins in evolutionary related clusters. Placing information about the domain architectures and gene neighborhoods onto the protein phylogenetic tree provides the means to understand evolutionary changes in proteins of known function and to facilitate functional prediction of uncharacterized genes.

There is a limited number of resources available for protein analysis in evolutionary context. Online version of MAFFT (9) gives an option to align sequences using iterative refinement method and to produce a Neighbor-Joining or UPGMA tree based on provided sequences. ClustalW2—Phylogeny and Simple Phylogeny (10) have an option to build a tree using a provided multiple sequence alignment (MSA). NGPhylogeny.fr (11) offers an option to construct MSA and build and visualize a phylogenetic tree; it allows a user to adjust parameters of each step and select a tree building program. IQ-TREE (12) is another online tool that builds MSA and a maximum likelihood tree. iTOL (13) enables combining a tree with the features identified in a set of protein sequences. The user needs to identify protein features and build a phylogenetic tree beforehand using external tools, and the data needs to be prepared according to the iTOL requirements. The STRING database (14) can produce gene neighborhoods for 4613 prokaryotic organisms (as of 2 November 2020). Protein sequences or identifiers from the same organism should be provided as input. STRING has no option for building a protein tree for evolutionary inference based on provided protein sequences. The online ETE Toolkit TreeViewer (15) combines tree and MSA in one picture. MSA can be presented as aligned blocks or in condensed format. Both, a tree and an alignment should be provided as input. TreeDom (16) allows a user to build a tree and map Pfam domains on it fetching the alignment and domain architectures from Pfam database. TreeDom is limited to building a Neighbor-Joining tree, as input PFAM-family identifiers should be used. TreeDom does not identify other protein features and does not provide an option for protein alignment. AnnoTree (17) enables visualization of precomputed genome annotations across the bacterial and archaeal phylogenies.

Here, we describe an intuitive platform, TREND, which has a capability to automatically identify protein features, calculate gene neighborhoods of each gene corresponding to provided prokaryotic proteins, cluster neighboring genes, identify operons, and put all these data in a phylogenetic context. To complete the full analysis, only a set of proteins sequences or protein identifiers is needed as input. The results are presented as an interactive picture on the web-site and can be downloaded for subsequent analysis. TREND is the only resource that can calculate gene neighborhoods for more than 125 000 prokaryotic genomes, identify protein features and correlate this with phylogeny in automated fashion, while providing ample opportunities for adjusting each step of the analysis.

## RESULTS

### Main features

**Figure 1.**
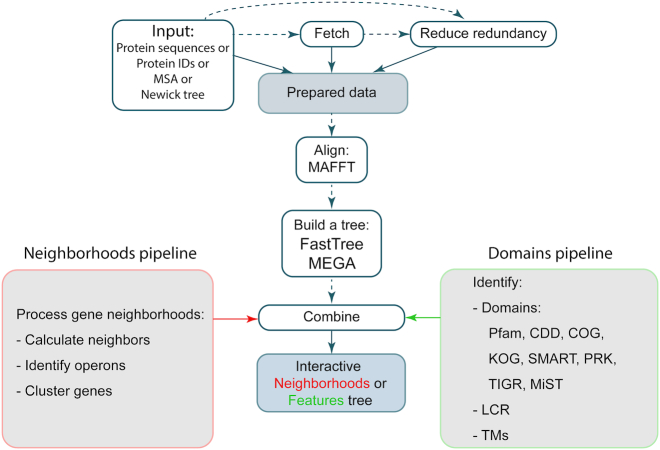
Domains and Neighborhoods pipelines in TREND. Steps that can be skipped are marked by dashed lines. If identifiers provided instead of protein sequences the sequences will be fetched from NCBI and/or MiST databases. Optionally the redundancy of the provided sequences can be reduced based on the identity level specified by a user.

TREND has an extensible modular architecture and consists of four main components: (i) a component dedicated to building a phylogenetic tree by running multiple sequence alignment and a tree building software; (ii) a component for identification of protein features and combining the results with the protein tree; (iii) a component for gene neighborhoods identification and mapping this information on the protein tree and (iv) a component for clustering genes and identification of operons. These components are grouped in two pipelines: Domains and Neighborhoods (Figure 1).

### Domains pipeline

A typical analysis starts with collecting similar protein sequences, for example, by running BLASTP against a protein sequence database using the protein of interest as a query. The collected set of protein sequences can be used as input for TREND (Figure 1). Another option is to input NCBI or MiST (18) compatible protein identifiers, such as RefSeq Id, locus tag or MiST stable Id, and corresponding sequences will be fetched from these resources. Upon the start, the pipeline will process input, identify protein features, build a tree, combine the results in one interactive picture, and present it to a user. A user can choose a database of Pfam profile HMMs (19) or CDD position-specific scoring matrices (20) for protein domains identification. We also integrated a collection of profile HMMs specific for signal transduction pathways in prokaryotes (18). Protein domains are identified by running HMMER (21) or RPS-BLAST (20) against the corresponding databases. In addition to protein domains, transmembrane and low-complexity regions are identified by running TMHMM (22) and SEG (23), correspondingly. Upon completion of this step, the sequences will be aligned using the algorithms implemented in MAFFT (9) and the tree will be built based on the MSA using FastTree (24) or MEGA (25). The user can customize each step adjusting corresponding parameters, choosing the alignment algorithm and the phylogenetic tree building method. The sequences in MSA will be sorted according to the order of the tree leaves, thus enabling the identification of amino acid residues that are conserved in specific, phylogenetically related groups of sequences. In addition, if a corresponding option is selected, the sequences in MSA and corresponding leaves will be enumerated. The pipeline also has a built in sequence redundancy reduction step implemented using CD-HIT (26), which will be executed, if the corresponding option is selected.

The domain analysis pipeline can be customized in a way that the tree will be built using specified parts of sequences, while protein features will be identified using full-length sequences. This is helpful when the evolutionary history of a specific domain or other fragment of sequences is explored. For this option, a user need to click ‘Add second area’ button and another input area will appear where fragments of sequences should be inserted.

If a user already has an alignment in the FASTA format, which was edited and prepared for the analysis, he can skip the alignment step and start the pipeline from tree building. If a user has a tree in the Newick format, it can be used together with the original sequences and (optionally) with an alignment, which was used to build the tree, to run the pipeline in a Partial mode. In this case, only the steps not related to tree building will be run. Instead of providing sequences used to build the tree, corresponding protein Ids can be placed at the beginning of the tree leaf names separated from the rest of the names by an underscore, a space, a forward slash or a vertical bar. In this case, the sequences will be fetched from NCBI or MiST. The Partial mode is particularly useful in domain evolution analysis and ancestral domain architecture reconstruction (27), in which case a prebuilt organismal phylogenetic tree can be combined with protein sequences containing a domain(s) of interest. TREND can also be used just as an online tree building tool if the protein identification step is skipped.

**Figure 2.**
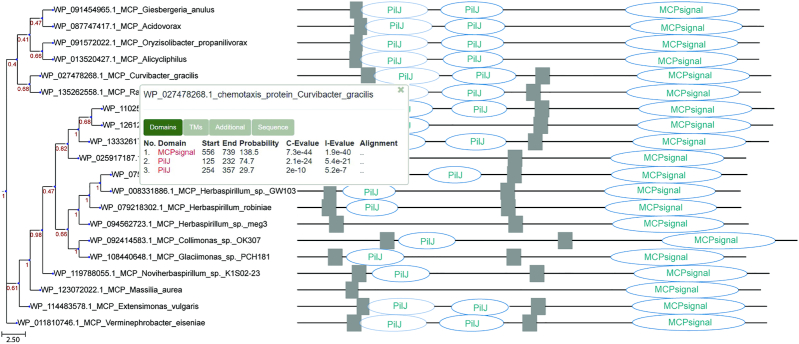
Phylogenetic tree of *Pseudomonas aeruginosa* PilJ chemoreceptor homologs combined with domain architecture (identified protein domains and transmembrane regions) generated by TREND. Domain nomenclature is according to the Pfam database.

As a result, an interactive tree will be produced (Figure 2). Clicking on features will open an information block with the details of the identified features. Domain analysis details contain links to entries in corresponding databases (Pfam and CDD) for each identified domain. Clicking on a feature will highlight the part of a sequence corresponding to the feature. The tree can be saved in SVG and Newick formats and used for further analysis. The identified features can be saved in JSON format for subsequent computational analysis, and the MSA—in FASTA.

### Neighborhoods pipeline

To start the gene neighborhoods pipeline, four types of input information can be used: (i) a set of protein sequences in FASTA format, (ii) an MSA in FASTA format, (iii) a tree in Newick format or (iv) a list of NCBI and/or MiST compatible protein identifiers separated by a new line character. The main prerequisite for the first tree types of input is the placement of protein identifiers at the beginning of corresponding protein names, separating them by space, vertical bar, forward slash or underscore from the rest of the names. Gene neighborhoods will be identified by fetching corresponding information from MiST database (18). Although any identifiers can be used to build the tree, it is required for the gene neighborhoods analysis to provide NCBI RefSeq identifiers, locus tags or MiST identifiers. If sequences or a list of Ids are provided, then the tree will be built based on the MSA constructed at the first step. If an MSA or a Newick-formatted tree was provided, then corresponding steps will be skipped. The type of the input is detected automatically and validated prior to running the pipeline.

**Figure 3.**
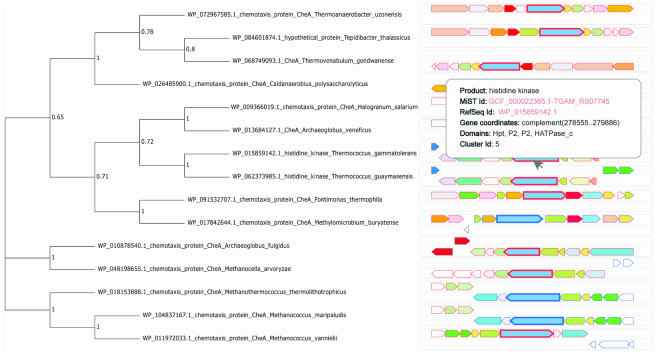
Phylogenetic tree of a subset of CheA kinase homologs and their corresponding gene neighborhoods generated by TREND. Genes that encode proteins consisting of the same domains are shown in the same color: *cheA* genes are shown in blue. Genes encoding proteins from corresponding branches of the phylogenetic tree (left panel) are marked with bold borders (in this case, *cheA* genes).

A user can specify the number of neighboring genes to be identified and processed for each provided protein. Upon identifying of gene neighborhoods, genes will be clustered based on shared domains of the encoded proteins. A user can control the permitted number of non shared domains for corresponding genes to be grouped in a cluster. In this case, not all domains in proteins should be shared for genes to be grouped. The next step is identification of operons based on the distance between the genes. The default distance between the genes is set to 200 bp (28). This parameter can be changed by a user. Similar to Domain analysis pipeline, sequences and corresponding tree leaves can be enumerated and sequence redundancy can be reduced. All this information will be combined and presented to a user as an interactive picture (Figure 3). All genes with the same set of domains, i.e. those that belong to the same cluster, are shown in the same color. Genes that belong to the same operon have the borders with the same color. Genes corresponding to the proteins that were used as queries are shown with thicker borders. Hovering the mouse over the picture shows the corresponding gene information including its product name, NCBI and MiST ids, links to corresponding databases, encoded domains and cluster ids. Gene neighborhood information together with the identified clusters and operons can be saved in JSON format and the picture can be saved in SVG format. Parsing the JSON formatted file allows a researcher to run an additional analysis, for example, to identify groups of gene clusters based on gene contents.

## CASE STUDY

To show how the usual computational workflow can be simplified using TREND we used it for the analysis of oxygen di-iron proteins (ODP) described in (29). In this work a new member of metalloenzymes superfamily (Pfam Lactamase_B PF00753) was identified, which was recruited to various bacterial and archaeal signal transduction (ST) pathways, including chemotaxis, to function as oxygen and iron sensors. The analysis done in that work identified several groups of ODPs: two groups of the sensors encoded in operons with methyl-accepting chemotaxis encoding genes in two different orders, ODPs in operons with different ST related genes, and the proteins fused with signal transduction domains. The computational workflow to make such an analysis would include running a set of tools to align proteins, build a tree, identify domains, identify gene neighborhoods for each gene, and then manually cross relating the generated data with extreme care in order to identify the groups and the events of domains fusion or detecting operons with ST proteins encoding genes. Applying TREND and interactively exploring the results we easily identified the branches on the tree (Supplementary Figure S1) with the described two types of operons (29) and the branches with signal transduction genes encoded in operons with ODP genes. Using Domains pipeline we identified domain architectures of the ODPs and observed the described groups of proteins fused with the signal transduction related domains (Supplementary Figure S1). Thus, TREND makes identification of genomic events more efficient allowing a researcher to automate all the necessary steps, and prevents the errors that can originate from the manual analysis.

## CONCLUSION AND FUTURE DEVELOPMENTS

TREND is a highly customizable computational platform that allows researchers to explore protein function identifying protein features, gene neighborhoods and operons, clustering neighboring genes, and integrating all these data into phylogenomic context and cross-referencing with RefSeq, Pfam, CDD and MiST databases. The extensible modular architecture facilitates adding new functionality with not much effort. The platform has a potential to grow into one of the major hubs for computational protein analysis and accelerate the research in computational biology. Among the future developments, we are planning to integrate Markov clustering of homologs and correlating the results with phylogeny, additional software for protein tree building and built-in BLASTP for collecting homologous proteins. We will be updating the web server by integrating new releases of Pfam, CDD and MiST databases and the underlying software when they become available.

## IMPLEMENTATION AND AVAILABILITY

The backend is implemented in Java using Spring Boot (https://spring.io/projects/spring-boot) and Hibernate ORM (https://hibernate.org/orm/) frameworks, PostgreSQL is used as a database system, Tomcat—as a web server, NGINX—as a reverse proxy. Separate scripts are written in Python and integrated in the application. Thymeleaf (https://www.thymeleaf.org/) is used as a template engine. The frontend is developed using jQuery, d3.js, Bootstrap and custom packages for data manipulation and visualization. TREND is freely available at http://trend.zhulinlab.org. GitHub repository location – https://github.com/ToshkaDev/TREND.

## Supplementary Material

gkaa243_Supplemental_Files
