# Correction to ‘TREND: a platform for exploring protein function in prokaryotes based on phylogenetic, domain architecture and gene neighborhood analyses’

**DOI:** 10.1093/nar/gkac034

**Published:** 2022-01-31

**Authors:** Vadim M Gumerov, Igor B Zhulin

**Affiliations:** Department of Microbiology and Translational Data Analytics Institute, The Ohio State University, Columbus, OH, USA; Department of Microbiology and Translational Data Analytics Institute, The Ohio State University, Columbus, OH, USA

The URL address of the web server published in this article has been changed from http://trend.zhulinlab.org to http://trend.evobionet.com/.

