# Supplementary material for: TREND: a platform for exploring protein function in prokaryotes based on phylogenetic, domain architecture and gene neighborhood analyses
Source: Nucleic Acids Res. 2020 Apr 13;48(W1):W72–6. doi: 10.1093/nar/gkaa243 (PMC7319448; doi:10.1093/nar/gkaa243)

FIg.S1. Phylogenetic tree of oxygen di-iron protein (ODP) homologs and their corresponding gene neighborhoods generated by TREND. ODP found in several types of neighborhoods encoding genes implicated in signal transduction (ST): in operons with kinases (Pkinase), methyl-accepting chemotaxis proteins (MCPs), di-guanylate cyclase/phosphodiesterase (GGDEF/EAL) and other ST domains (START, HD). Two types of operons encoding ODP and MCP in different orders are marked by rectangles. MCP in orange rectangle has a distinct sequence pattern.

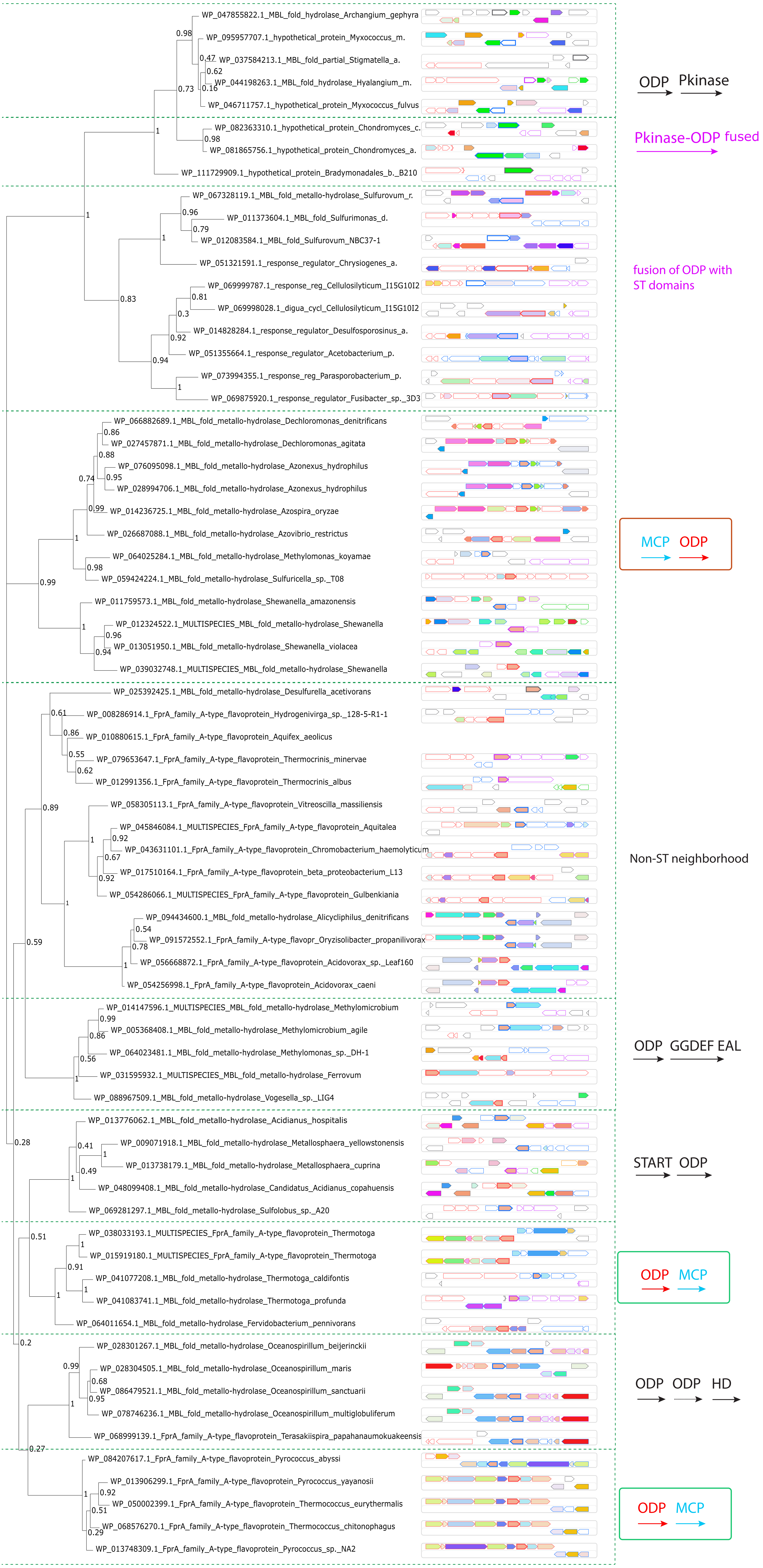

Supplement: gkaa243_Supplemental_Files [file gkaa243_supplemental_files.zip › Fig.S1.pdf]
