# Supplementary material for: TREND: a platform for exploring protein function in prokaryotes based on phylogenetic, domain architecture and gene neighborhood analyses
Source: Nucleic Acids Res. 2020 Apr 13;48(W1):W72–6. doi: 10.1093/nar/gkaa243 (PMC7319448; doi:10.1093/nar/gkaa243)

Fig.S2. Phylogenetic tree of oxygen di-iron protein (ODP) homologs combined with domain architecture generated by TREND. ODP homologs grouped in separate branches as one-domain proteins in signal transduction (ST) related and also non ST gene neighbrohoods and as fused with signal transduction domains in multi-domain proteins. MCP - methyl-accepting chemotaxis protein, Pkinase - Protein kinase, START - StAR-related transfer domain, GGDEF - di-guanylate cyclase, EAL - di-guanylate phosphodiesterase, HD - metal-dependent phosphohydrolase.

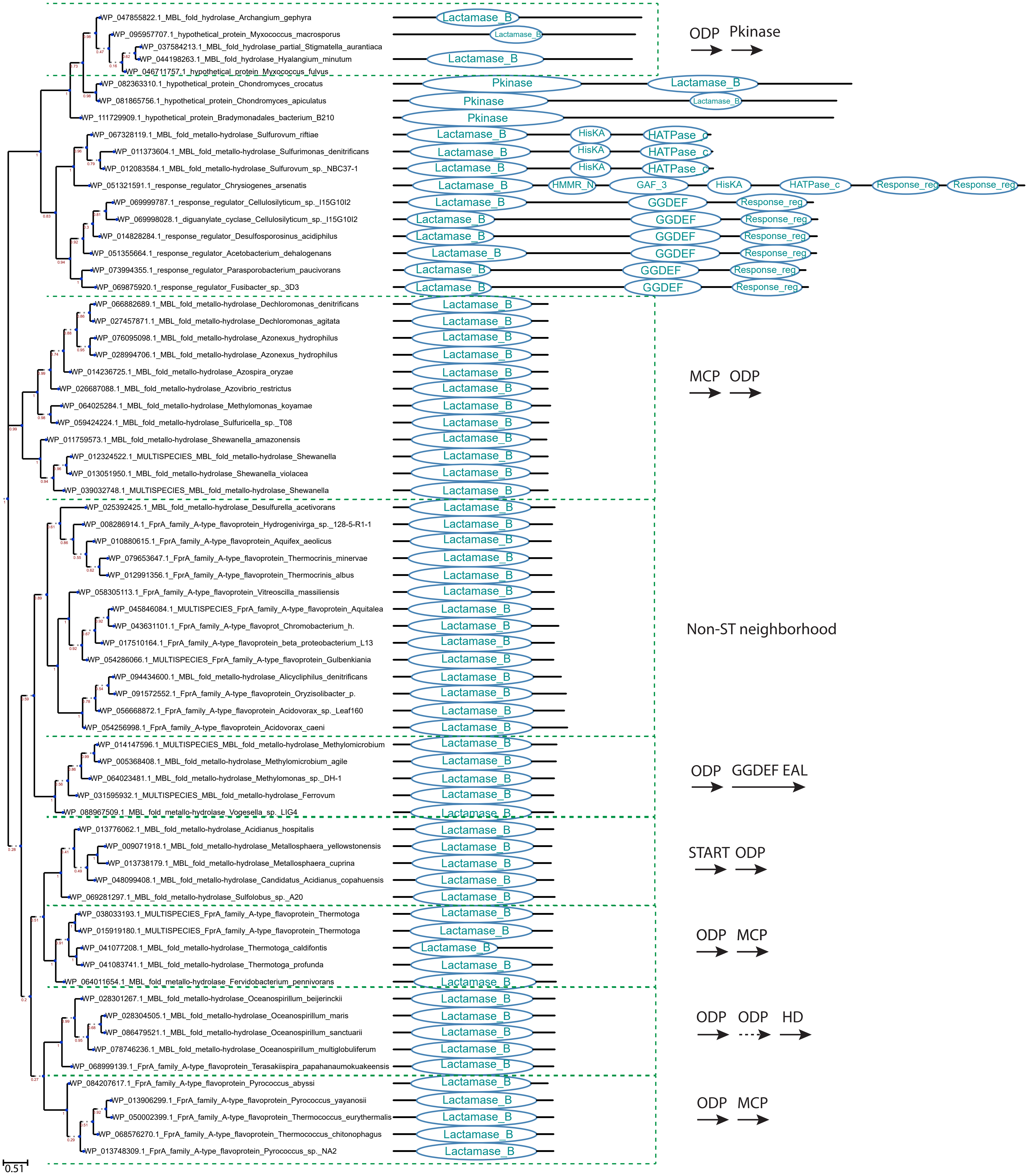

Supplement: gkaa243_Supplemental_Files [file gkaa243_supplemental_files.zip › Fig.S2.pdf]
